# Evaluation of computerized health management information system for primary health care in rural India

**DOI:** 10.1186/1472-6963-10-310

**Published:** 2010-11-16

**Authors:** Anand Krishnan, Baridalyne Nongkynrih, Kapil Yadav, Satyavir Singh, Vivek Gupta

**Affiliations:** 1 Centre for Community Medicine, All India Institute of Medical Sciences (AIIMS) New Delhi, India; 2 Media Lab Asia, New Delhi, India

## Abstract

**Background:**

The Comprehensive Rural Health Services Project Ballabgarh, run by All India Institute of Medical Sciences (AIIMS), New Delhi has a computerized Health Management Information System (HMIS) since 1988. The HMIS at Ballabgarh has undergone evolution and is currently in its third version which uses generic and open source software. This study was conducted to evaluate the effectiveness of a computerized Health Management Information System in rural health system in India.

**Methods:**

The data for evaluation were collected by in-depth interviews of the stakeholders i.e. program managers (authors) and health workers. Health Workers from AIIMS and Non-AIIMS Primary Health Centers were interviewed to compare the manual with computerized HMIS. A cost comparison between the two methods was carried out based on market costs. The resource utilization for both manual and computerized HMIS was identified based on workers' interviews.

**Results:**

There have been no major hardware problems in use of computerized HMIS. More than 95% of data was found to be accurate. Health workers acknowledge the usefulness of HMIS in service delivery, data storage, generation of workplans and reports. For program managers, it provides a better tool for monitoring and supervision and data management. The initial cost incurred in computerization of two Primary Health Centers was estimated to be Indian National Rupee (INR) 1674,217 (USD 35,622). Equivalent annual incremental cost of capital items was estimated as INR 198,017 (USD 4213). The annual savings is around INR 894,283 (USD 11,924).

**Conclusion:**

The major advantage of computerization has been in saving of time of health workers in record keeping and report generation. The initial capital costs of computerization can be recovered within two years of implementation if the system is fully operational. Computerization has enabled implementation of a good system for service delivery, monitoring and supervision.

## Background

A health management information system (HMIS) is a process whereby health data (input) are recorded, stored, retrieved and processed for decision-making (output). Decision making broadly includes managerial aspects such as planning, organizing and control of health care facilities at the national, state and institution levels [1]. In public health programmes HMIS would be primarily concerned with health care delivery issues like - antenatal care, immunization, and disease control programs and administrative issues like reporting, inventory management, financial management, and vehicle and personnel management issues. Therefore maintaining a good HMIS is an essential part of running a health system. This can be done manually as it is being done in most of India, or it can be maintained in a computerized system.

Though, India has a vast network of Primary Health Centers (PHC) to provide primary health care in rural areas, its functioning has been suboptimal. In order to function efficiently, health managers need to do regular monitoring of the health status of the population, medicines, vaccines, requirement and utilization patterns, equipment availability and status etc. Timely and accurate information is necessary to formulate and implement effective programmes. Such information is currently lacking in the health system in the country. Health workers generate lot of data in the village, send it to the PHC, where it is compiled in the form of monthly reports and transferred to the secondary level. Primarily the flow of data is unidirectional. In addition, a lot of data that the health workers collect is redundant or never utilized adequately. Efficient management of data is difficult in a manual system, and often involves duplication of efforts and wastage of time. A computerized management information system is one among the many ways that Information Technology (IT) can help improve the health system; IT can aid the workers in providing services, data collection, storage, analysis and dissemination of information.

There have been very few examples globally where a computerized HMIS has been operational in a population based health care delivery system for a significant duration [1-3]. In evaluation of a Health Management information system in Uganda, based on interviews with doctors and nurses, the authors concluded that introduction of HMIS resulted in health workers valuing the data generated by them better; it supported program planning and decision making as well as improved the quality of and access to health care [4]. Many of the evaluations have also looked at the utility of HMIS as a tool to assist organizational development [5,6].

In this paper we share our evaluation of the HMIS which has been operational in a rural health system in Ballabgarh for about two decades now. The research questions being addressed in this study were: What are the benefits of a computerized HMIS as compared to a manual system, and what are the issues which need to be considered to upscale to a higher level.

## Methods

### Background of the project area

The Comprehensive Rural Health Services Project (CRHSP) Ballabgarh was started in 1965 and is situated in Ballabgarh, Haryana in Northern India. It is run by the All India Institute of Medical Sciences, New Delhi in active collaboration with the State Government of Haryana with the objective of demonstrating a model health care delivery system and for training of medical students. The Intensive Field Practice Area of the project comprises of 28 villages catering to a population of 85,552 in the year 2008. The health care in these villages is provided according to the national pattern. There are two PHCs under the Project, namely-Dayalpur PHC and Chhainsa PHC with six subcenters each. The staff consists of medical officers, 24 health workers (each subcenter has one male and one female health worker) and four supervisors. As in all the PHCs throughout the country, all activities related to national health programmes are implemented through the health workers. The health workers (male and female) make house visits every fortnight in their respective subcenters. Besides, male workers are also responsible for registration of vital events. Apart from the routine continuous collection of demographic information, annual census is conducted in the month of December by the male workers. Information in about 10% of households is crosschecked by the supervisor and another 5% by the medical officers for completeness and accuracy. In order to overcome the problems faced by the health workers in handling data and making efficient use of data collected, a computerized data management system was introduced in the Comprehensive Rural Health Services Project Ballabgarh in February 1988 [3]. The full computerization was completed in 1990.

### Description of HMIS

Haazen *et al *have advocated a tri-axial framework for HMIS comprising of system dimension, system uses and system sectors/stakeholders in terms of its placement in a system hierarchy [7]. However we use a simplified framework for describing our HMIS in terms of the technology, human resources and the output of the system.

#### Technology

**Figure 1:**
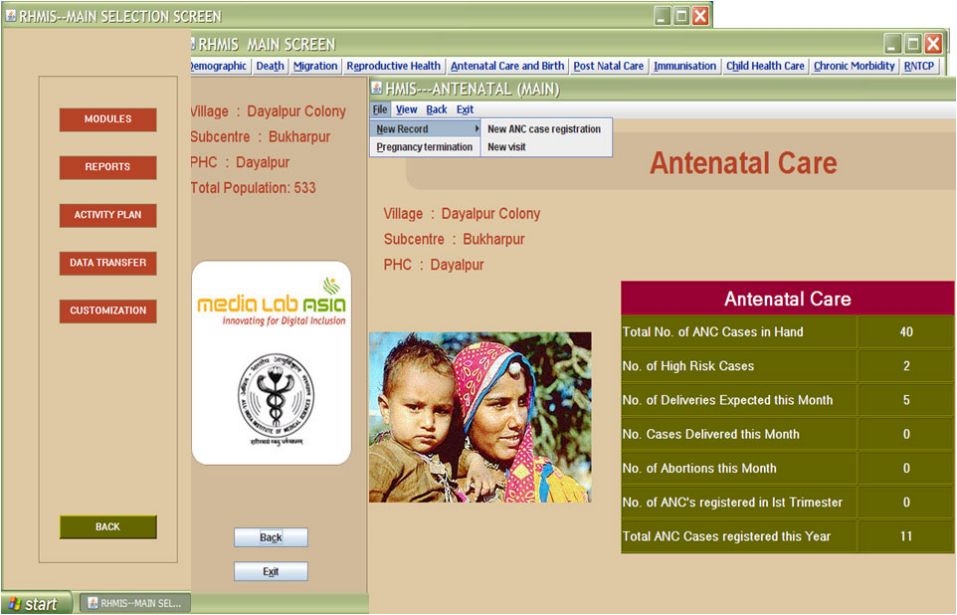
**Screenshots of HMIS user interface showing the Main Selection, Main Modules and Antenatal Module screens**.

Based on our experience, these can be broadly divided into the software being used and the hardware on which the system is operational. The current hardware running the HMIS is Intel Pentium IV, 1.60 GHz processor with 256 MB RAM. The software can be described in term of the core database, the user interface and the operating system (OS). The HMIS database has a relational design. The data is stored in 86 tables which can be broadly divided into the following types: (1) Data-entry tables, where data of different modules such as demographic, Antenatal care (ANC) etc. are saved (2) System tables where the codes for village, education, caste and other variables used in the program are stored (3) Archive tables, where old data after events such as pregnancy termination (ANC data), death (demographic data) are maintained (4) Report tables where reports are stored temporarily for printing. Every individual in the project area has been assigned an identification number which is also used as a primary key in the database. Fresh births and in-migrants are automatically assigned new primary keys. The user interface of the HMIS is menu driven, easy for non-computer professionals to use. User guidance is available through tool-tips and messages. The menu system is modular, each module catering to specific health services provided in the area. Some of the modules are demographic module, antenatal module, death module etc (Figure 1). Being a relational database, data entry through one module automatically updates the linked fields in other modules. The interface includes consistency checks for data quality, drop down menus for simplifying data updating and provision for addition of variables. The operating system is Microsoft Windows XP Professional Edition.

The HMIS has undergone three generation evolution over the years reflecting the advancement in information technology as well as changing perceptions of the users of HMIS (Table 1). For the current version, MySQL was chosen as it can handle large database, is stable, secure, scalable and free. It can also act as server allowing multiple users to simultaneously access the database. Java (J2SE) programming language was chosen for development of a graphical user interface (GUI). Another major reason for choosing MySQL and Java was their platform independency i.e. they can run on any operating system like Windows, Linux, Macintosh, Solaris etc allowing flexibility. Databases for each PHC are on separate computers as fetching during data entry were noted to be time consuming with a combined database. The hardware is housed at its project headquarters at Ballabgarh and not at PHCs due to logistic reasons.

**Table 1:** Evolution of technology used in Ballabgarh HMIS

	Phase 1	Phase 2	Phase 3
Features	1988-2002	2002 - 2005	2006 (Generic HMIS)
Operating system	MS DOS	Windows	Linux/Platform Independent

Software(Front end)	FoxPro	Visual Basic	Java

Database(Back end)	Dbase III	Microsoft Access	MySQL

#### Human Resources

A data entry operator updates the data with the assistance of the health workers once a month. Thus, currently the data entry operator spends two weeks for this purpose. On other days, he works with hospital data.

#### Output

**Figure 2:**
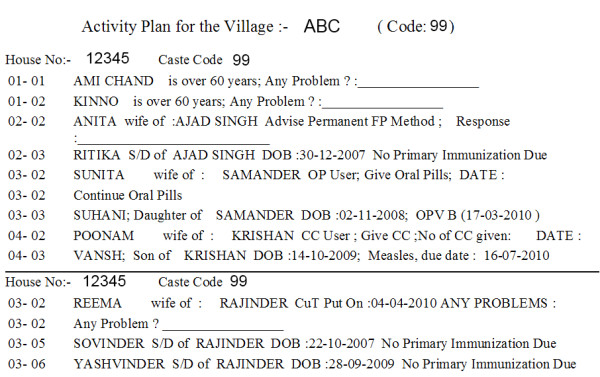
**Sample output from the Ballabgarh HMIS: Workplan**. Abbreviations: DOB - Date of birth. FP - family planning. OP - oral pill. OPV-B - Oral Polio vaccine Booster. CC - condom. CuT - Copper T.

The main output of the HMIS is the *workplan *generated each month after the data has been updated. The *workplan *lists the monthly activities by house and contains updated information about all the individuals including the under-five children, pregnant women, eligible couples, and geriatric age-group in the house. It has a list of specific health services that need to be provided for each individual (Figure 2). The *workplan *also serves as a tool for monitoring of the workers by the medical officer and the supervisors. Other HMIS outputs include monthly reports, lists for immunization and contraceptive services, and performance indicators of workers, subcenters and PHCs. Annual performance review of each worker is done based on the indicators generated from the HMIS.

### Framework of Evaluation of HMIS

An evaluation of the HMIS can only be done in the context of the objectives of its development. The objectives, functions and outputs required out of the HMIS are listed in Table 2. The primary purpose of HMIS was to support health workers in delivering health care services to the population. The other important objective was to support program managers in monitoring and supervision of the workers. In this context, the evaluation of the HMIS was based on the framework shown in Additional file 1: Table S1. This evaluation framework was adapted from the Performance of Routine Information System Management (PRISM) framework which uses a systems framework of inputs (technical, organizational and behavioral factors), processes, outputs, outcomes and impact [8,9]. The evaluation was mainly formative and from the user's perspective. Littlejohns P et al have also listed certain evaluation criteria which are consistent with this current evaluation [2]. Odhiembo-otieno et al have listed the evaluation criteria for HMIS based on their experience in Kenya, and divided them into three phases: pre-implementation, concurrent and post implementation phases [10]. The indicators used in this paper are consistent with the concurrent and post-implementation phases proposed.

**Table 2:** Programmatic features of Ballabgarh HMIS

Objectives	Functions	Outputs
1. Data Storage	• Hold Family Case Records • Data on Service provision records like immunization, Family Planning etc.	• Database

2. Improve Service Delivery	• Generate work plan • Generate eligible lists • Generate census list	• Work Plan • Eligible list • Census List

3. Program Monitoring	• Generate Worker's annual performance • Generate List for random checks • Generate annual service coverage statistics	• Worker Annual • Performance score • Random list • Annual Report

4. Improving Reporting	• Generate Monthly reports	• Monthly report

5. Support Research Activities	• Provide a sampling frame • Provide data for analysis	• Publications • Theses • Research Projects

The World Health Organization (WHO) has given certain guidelines under which evaluation of HMIS is to be done i.e., data generation and report compilation, data utilization, details about computer hardware and software, training and monitoring [11]. This evaluation is also in tune with the WHO guidelines.

### Methodology of data collection to compare manual and computerized HMIS

The data for evaluation were collected by in-depth interviews of the program managers (authors) and health workers. In depth interview method was chosen because, unlike focus group discussions, it gave the opportunity for one- to- one interaction, and individual opinions could be elicited. Health workers from AIIMS (using computerized HMIS) and non AIIMS centers (using manual HMIS) were interviewed to identify the benefits of computerization.

As described earlier, the male workers are involved in vital events registration, so we decided to interview only the male workers for this study. Male workers from all the 12 subcenters under AIIMS were included in the study. During the period of data collection, one worker was not available so finally only 11 male workers from AIIMS were interviewed. Besides our PHCs, there are 3 other PHCs in the same district, which are not under AIIMS. Correspondingly 11 male workers from these adjacent non-AIIMS PHCs were selected Therefore a total of 22 health workers were selected for in-depth interviews. With the exception of computerization, both these sets of workers were expected to carry out similar activities under the various national health programs, and work under similar conditions.

Interviews were also conducted with two medical officers of the PHCs and two program managers (authors). The interviews for program managers and medical officers were carried out only for the staff under AIIMS since this study was primarily planned to assess its usefulness at the user's (Health workers) end and to identify problems and areas which need strengthening at the grass root level.

Data collection was carried out in May 2009. This was a descriptive study and a semi-structured in-depth interview guide was used as study instrument. The study instrument had questions under different domains such as record keeping, data quality, supervision and feedback and overall usefulness of the computerization of HMIS (Appendix).

#### Analysis of Qualitative Data

The interviews were recorded in a voice recorder. A coding framework was prepared based on the interview schedule. Transcription of data was done in Hindi (local language), and later translated to English. The transcripts were read and responses were free listed; most commonly occurring codes were identified. The codes identified were grouped in specific categories based on the pre-identified themes in the evaluation framework. The analysis was done manually and no software was used for analysis. The analysis was done by two researchers (AK, KY) separately and compared. In case of a discrepancy, the issues were discussed and resolved by consensus.

#### Cost Analysis

A cost assessment was conducted using the standard procedure of identification, measurement and evaluation of cost. The objective was to estimate the incremental cost of starting a computerized system in place of the existing manual system. This was adopted as incremental costs were most relevant from the point of a program manager wishing to changeover to the new system. The other outcome measure for cost analysis was cost per individual. The comprehensive identification of the costs involved in the HMIS was done through discussions with the staff working in the HMIS team. All the authors have been involved in the development and running of the HMIS for at least seven years with the lead author being involved for last twenty years. The costs were classified into two broad categories:

(1) Capital cost - The capital cost consists of those items which have a life of more than one year and represent an initial investment. Training cost and software development was treated as a capital cost with life of ten years. Data transfer was also considered as a one-time investment with a life of 20 years. No space or building cost was included as existing health facilities would be able to accommodate the requirement within the available space. The capital costs were converted to *equivalent annual costs *based on their useful life years and a discount rate of 5%.

(2) Recurrent costs (consumables & salaries) - Recurrent costs include those items that have less than one year of life and largely consisted of human resource cost and cost of consumables like paper, cartridges, electricity etc. Two set of computers and printers were estimated to be required to house the database and facilitate easy working. The time required for training and database transfer was estimated based on our experience of doing these activities. The time spent by workers in planning their work, record keeping, report preparation at subcenter level, compilation at PHC level as well as review by medical officer were based on interview with workers of AIIMS and non-AIIMS. The costs of maintenance, stationery, electricity were based on actual as recovered from records. The cost of all equipments including computer and printer was based on market rates for 2008. The cost of time spent by all human resources was estimated based on their current salary structure under Government of India.

The difference between the two systems of data storage gave *the incremental cost *of shifting to the computerized system. Dividing the total cost with 85,552 (population of the project area in 2008) gave the *cost per individual*.

### Ethical issues

This paper presents the results of a program evaluation by the program managers and implementers themselves with the purpose of learning lessons for themselves and sharing with others. This was considered as a routine part of the job of the authors of the paper and hence no separate ethical clearance was obtained. Written consent was taken for interviews with the field workers. Most the information derived for costing was based on internal discussions of the team and no formal interviews were held.

## Results

Based on the interviews with different stakeholders and experience of the authors the results are presented as per the framework. The in-depth interviews were audio recorded and transcribed. The duration of the audio recordings was about fourteen hours and about fifty three pages of transcripts were generated.

### Inputs

#### Technical Support

The technical support to the HMIS was initially provided by Media Lab Asia, Department of Information Technology, Government of India, and subsequently has been taken over by the AIIMS team.

##### Hardware maintenance

There has been no major hardware problem since computerization. While there are two computers (one for each PHCs), these are maintained at Ballabgarh, and have not been shifted to PHCs due to poor logistic support in terms of electricity supply and difficulty of accessing hardware engineers to provide service in rural areas.

##### Software maintenance

Minor problems in software continue to arise requiring some programming modifications. These are regularly taken care of by the AIIMS team without need for any external assistance. A monthly meeting is held to review the working of HMIS and any problems identified are discussed and solutions identified.

#### Organizational

##### Training

The current technical staff has been trained in use of computerized HMIS at the time of its installation. A training manual has also been prepared.

##### Finances

There is no separate budget for the HMIS. The entire funding for this program comes from AIIMS under the budget head for the rural health program. All the staff, infrastructure, resources are inclusive of the whole program, and not especially for HMIS. This can be interpreted in a positive aspect wherein the cost of HMIS is adjusted within the existing facilities without any special budget allocation.

##### Sustainability

The fact that a computerized system has been in place for the last twenty years is in itself a testimony to its sustainability. A clear understanding of the potential benefits, strong commitment from the team at Ballabgarh and appropriate technical support are responsible for this sustained use. The fact that the system has undergone modification regularly to cater to the demand of the health system has ensured the continued relevance for the computerized HMIS.

### Processes

#### Data flow

Data are collected at the subcenter level by individual health workers. Additions or modifications are noted in the workplan as they carry out their activities. At the end of the month the workers update the HMIS in CRHSP with the assistance of a data entry operator. After every monthly update, *workplan *for the subsequent month is generated and handed over to the workers; therefore monthly updated information is available. Besides the workplan, a *monthly report *is generated and submitted to the District level. Consequently, at the end of the month, the workers get a feedback of the previous month's performance and the problems and possible solutions are discussed with the supervisors.

#### Data Security

Since the database stores personal information on a large population where they can be identified by names, data security is a vital concern. A security audit of the system has not yet been conducted. Still, to maximize data security, several steps have been instituted. The hardware and software setup is access controlled with multiple passwords (boot time, HMIS application, and database). All data access by personnel other than data entry operators are logged in a register. Regular weekly and monthly backups are taken on optical disks which are stored in lock and key. Anti-virus software is installed on the machines and they are regularly updated using offline techniques. To minimize the risk of hacking, the HMIS computers have not been connected to internet or local area network. The PHC medical officers are responsible for the authenticity of the data entered in the HMIS. For data integrity, each time a row in the database tables gets updated; information on time and the person who has updated gets logged in the database. However it does not store the previous values. To ensure application integrity, any changes in software code of the application or the database, are reviewed with the project in-charge before being implemented. The primary key is not included in any of the outputs of the HMIS. There is a policy on data sharing with researchers (both AIIMS and non-AIIMS). If any HMIS data is to be utilized for research activities, informed consent is taken from the individuals prior to utilization of the data. Logs are maintained for all data sharing requests.

### Outputs

#### Data quality

The reliability of data is often a concern many experts have voiced about field data from developing countries. Computerization ensures that vital events registration is more accurate and timely. It improves record keeping, integrity and validity of data through better supervision and a possibility of a random check. In our HMIS, there is an inbuilt system to crosscheck the completeness and accuracy of data. Every year the annual census is carried out in December, where 100% verification of data is done, and we have found that there are no major discrepancies in the data. The reliability and validity of data is also verified by external users - either investigators of research projects or postgraduates of Centre for Community Medicine e.g. in an ongoing project at CRHSP, research workers collected demographic data and it was identified that in more than 95% of the fields, the demographic information in the HMIS was accurate (Broor S. *Direct and Indirect Protection by Influenza Vaccine given to children in India*. *Project NI-1099, All India Institute of Medical Sciences*. Department of Microbiology, All India Institute of Medical Sciences; funded by Centre for Diseases Control, Atlanta, USA, Ongoing project). Also, in 2009, an exercise to validate the database was done by checking the total births in the project area in a sample of 100 infants (This was done as a part of a thesis to find out the determinants of institutional deliveries). It was observed that in one case the gender of the child was not correct; otherwise there was no other discrepancy [12].

#### Information use

##### Better data storage and management

Paper based records at PHCs were prone to termite attacks and would need regular renewal. While, initially at Ballabgarh data were stored on floppy discs and then Compact Discs (CDs), better storage options are now available. Data processing is faster, and retrieval of data could be done rapidly. Workplan and generation of list of eligibles, is a productive feedback that saves workers' time and energy.

"(Computerized) HMIS also helps us in maintaining our records properly. The Family Record Card (FRC-generated annually by HMIS before annual census) helps us a lot during the annual census. We are able to track every birth and death in our field by cross checking with the FRC. Eligible couple list generated by HMIS also saves lot of our time, which we otherwise would have to spend to update this list from our registers."

-Health worker from AIIMS area

*"If suppose in the future, any officer comes and asks for records. To get the information from register, it would take time. But by computer, we can do it immediately*.

- Health worker from non-AIIMS area

##### Improved Monitoring and Supervision

It has provided a better tool for monitoring and assessing the health worker's performance in a more objective manner. The recording and listing of activities in Workplan makes supervision easier. A six monthly review of program performance using indicators generated from HMIS makes the workers understand the need for the data collected and also to take timely action if needed. HMIS also allows a generation of workplan for supervisors and medical officers. This includes a randomly generated sample of the previous months work entered in to the system for verification e.g. details like immunization, births and deaths, contraceptive users. It creates a system for quality control. A health worker performance report can also be generated on monthly basis.

"We have been using HMIS for monitoring and supervision of health workers for a long time. We have developed certain indicators like percentage of Antenatal care (ANC) registered within 12 weeks by the health worker, to objectively measure the performance of individual health workers. These indicators are generated from HMIS and are then used to fill the Annual Confidential Report (ACR). This objective and transparent evaluation system is much appreciated by the health workers."

- Program Manager, CRHSP area

##### Use for research purpose

While the primary purpose of HMIS, was and should be, related to improving health care service delivery, its potential for research is also immense. Another offshoot of the computerized HMIS was that it could be used in research activities, since accurate data were available for the entire population and over a long period of time. Some of our research publications from the HMIS illustrate this potential [13-17]. Only in the recent years, the research potential has been realized. The database is currently being modified to address possible research issues. Availability of a large community based cohort with computerized HMIS makes this centre attractive for vaccine trials. We have also used HMIS for the training of postgraduates.

"For example if I want to get a list of all infant deaths that occurred in Ballabgarh in last 5 years and study its association with various determinants, HMIS can provide me with the required information within minutes. To extract similar information from manual registers will take days and months or may not be possible at all. The longitudinal database of HMIS has immense research potential"

- Program Manager, CRHSP area

### Outcomes (improved system performance)

#### User Perspectives of advantages and disadvantages

A summary of the workers' responses have been presented in Table 3. Some of them are summarized below:

**Table 3:** Comparison of health workers' interviews on the manual versus computerized HMIS

Issues	Non-Computerized system	Computerized System
Record keeping: (Number of registers, time taken and problem)	• Registers: 7 - 15 • Average time: *around 2 hrs *(0.5 - 3 hrs.) • No major problem identified	• Registers: 7 - 10 • *1 hour *(0.5-1.5 hrs) • No major problems

Estimation of target population e.g. the eligible children for vaccination, or number of antenatal cases	• Procedure for estimation not known as targets are provided from District level.	• Estimation is done from information available in HMIS

Calculation of estimation of Immunization coverage and backlogs	• Based on monthly report and immunization register. • Backlogs are identified with the help of ASHA1, AWW2	• Based on monthly report, and immunization register • Backlog by Work plan (eligible list) and HMIS.

Reported completeness of Birth/Death Registration	• About 95% (90 - 100%) verified during field visits.	• All (100%) verified during field visits and during census.

Updating of records after Annual Census	• First, overwrite in the old register. After that they update in new register. • *Takes about 2 months (1 - 3 month)*	• Make changes in the paper print out of families and update the computerized database. • *Takes about 3 days (2-4 days) *for each subcentre.

Preparation of monthly report (Process and time)	• Compile data from various registers • Takes about *3 days (2 to 5 days)*	• Compile from workplan and computer generated information • Takes about *6 hours *(*2 hours to 1 day)*

Feedback from higher levels (CHC/District hospital/Ballabgarh) on the monthly reports	• *No feedback *from Community Health Centre/District Hospital. They discuss the issues during monthly meeting.	• They *get monthly feedback *and discuss the issues during PHC monthly meeting. • They also get work plan for next month from Ballabgarh

Perceived benefits of computerization of records	• Not Applicable	• *Work plan makes the work very easy*. They know what to do during house visits. It reduces time spent per house. • Senior officers can monitor the field performance of health workers objectively.

^1 ^ASHA - Accredited Social Health Activists, ^2 ^AWW - Anganwadi workers. These are grass root level workers from the community who are responsible for mobilizing the community to avail maternal and child health services.

##### Time saving

The major advantage was in the time saved by the computerized system. The workers took double the time to update their records in a manual system as compared to a computerized one (average time taken was about 2 hours in a manual system as compared to 1 hour in the computerized one). Similarly, after the annual census, it took about 3 months to update the records in the manual system, as compared to 3-4 days in the computerized system.

##### Improving Service delivery

A computerized HMIS has improved service delivery and has helped the workers during home visits e.g. reminders generated for ANC check up, Immunization, follow-up of family planning acceptors. The work-plan generated after each update serves as a guide for health workers' activity in the field. Work plan is also used by the health workers for data recording during their daily house visits.

"Computerization is very useful; it has made working in field easier. The monthly work plans tells us even before we enter the household that what work we are supposed to do in that particular household. Also work plan helps us in planning our daily activity and we can take necessary medicines etc before we leave the sub centre and go to field as per the activities listed out in work plan."

-Health worker from AIIMS area

*"We identify the immunization backlogs from our register and ask the ASHAs (*community health workers*) to call those children to the subcenter. During survey, we correct the data in the old register and rewrite the corrected data in a new register*.

- Health worker from non-AIIMS area

##### Report Generation

It has aided in report generation monthly and annual reports could be generated easily and rapidly. It was also possible to address loose end queries or generating unscheduled reports besides the routine e.g. information about measles immunization in any village in case of a measles outbreak etc.

#### Program coverage indicators

There has been a consistent high coverage with childhood vaccines and antenatal care in the population for the last fifteen years (Table 4). Availability of a computerized HMIS has resulted in better monitoring of health indicators. High coverage could be partially attributed to better supervision and monitoring. Therefore a functional HMIS has played an important role in running and maintaining an efficient system.

**Table 4:** Health service delivery indicators for major national programs since 1990 in CRHSP Ballabgarh & comparison with indicators of Faridabad District

Indicator (%)	1990	1995	2000	2005	Faridabad District (2002-04)*
Antenatal Registration	Not available	93%	95%	95%	36.3%

Coverage of antenatal with tetanus toxoid	Not available	90%	93%	95%	80.1%

Deliveries by Skilled Birth Attendants	4.4	4.6	20.9	30.0	31.2%

Couple Protection Rate	41	40	44.5	59.4	47.9%

Immunization Coverage (12-23 months)					
BCG	96	92	99	98	75.4
DPT3	91	82	99	98	60.4
OPV3	95	84	99	98	62.0
Measles	81	72	99	98	61.1

* District Level Household and Facility Survey-2, Haryana; Ministry of Health and Family Welfare Government of India 2002-04 [25].

### Cost evaluation(Table 5)

**Table 5:** Annual Cost (INR) of functioning manual and computerized Ballabgarh HMIS

	Item	Computerized System	Manual System	Incremental Costs (Computerized system - manual system)
**Capital**		**198017**	**0**	**190817**
	Computer cost (2)	16168	0	
	Printer Cost (2)	6929	0	
	Training Cost	11008	0	
	Software development	129505	0	
	Data Transfer cost	32097	0	
	Furniture	2310	0	

**Salary**		**1432800**	**2563200**	-**1130400**
	Workers Time	1252800	2563200	
	Data Entry Operator	150000	0	

**Consumables**		**43400**	**5300**	**38100**
	Maintenance Cost of hardware	10000	0	
	Stationery/cartridges	27400	5300	
	Electricity	6000	0	

**Total**		**1674217** **(35,622USD)**	**2568500** **(54,649USD)**	- **894283** **(11,924USD)**

The initial cost incurred in computerization of two PHCs was estimated to be INR 1,674,217 (USD 35,622 @ INR 47 = 1 USD). The equivalent annual incremental cost of capital items was estimated as INR 198,017 (USD 4,213). A major saving of computerization was seen in the time saved by the workers in record keeping and report preparation. This is in keeping with the results of interviews with workers mentioned above. The amount annually saved due to computerization of the HMIS came out to be around INR 894,283 (USD 11,924). In other words, the original capital investment was recovered within two years. This is provided of course, that the time saved by health workers due to computerization is utilized effectively for program delivery. The annual cost per person was estimated to be INR 18 for the computerized system as compared to INR 28 for the manual system.

## Discussion

Our experience of two decades with a computerized system resulting in a high service coverage statistics indicate the feasibility and desirability of implementing such a system for strengthening the health care delivery system in rural India. We have seen that our system has achieved most of what it set out to achieve in terms of improving the service delivery. The chief concern of the program managers was always of workload of health workers and quality of data at the level of generation of the data. We have demonstrated that these concerns can be addressed and would result in a more effective and efficient system. In fact, in our centre, we are moving further ahead with contemplating the use of handheld computers by the health workers [17]. However, currently, the computerization stops at the secondary level i.e. CRHSP Ballabgarh level, and there is no link with higher facilities as these are not computerized. Also, currently, the HMIS does not cover other important needs such as inventory management.

The need for strengthening information systems in health care and the role that information technology can play in this has been emphasized by many authors [18-23]. Lucas et al have also identified that one of the ways that IT can improve future health systems is to improve traditional health information systems [18]. A need for strengthening information systems and focus on building local capacity, increase utilization of data for planning and decision making has also been pointed out by Adindu et al [19]. Lack of denominators for immunization coverage assessment and target setting result in inability to assess performance of workers was also identified by Braa et al [20].

Most studies on Health Management Information Systems actually deal with three types of systems. The most common is *electronic medical records *of patients. The second one is about a software for compiling aggregate reports. The third type is a name-based system which can generate monthly reports as well. Our HMIS is an example of the third type i.e. a name based system. An experience of using an electronic HMIS for public health information in three districts of Himachal Pradesh, India in collaboration with German Agency for Technical Collaboration has also been positive [21]. However, that was not a name based system but only reported information from the block level with linkage till state level. The front end was in Visual Basic and back end in MS Access. The computerization saved workers time in keeping multiple registers. Visual display of analysis of report improved managerial decision making. The challenges identified were maintenance of software and skeptical users at field level.

Our results mirror the experience of another experiment in India on a development of computerized HMIS to support the delivery of public health programs for MCH services using a modular approach. Moidu and Singh et al have demonstrated an improvement in delivery of MCH services (ANC and immunization) following an implementation of a computer-based information system in India [22,23]. They also showed that in economic terms, the cost of a fully immunized child was reduced with reduction of dropouts - improved cost effectiveness. This and other national experiences described above clearly highlight that there is a need to upscale such a system to national level.

We have compared our health service indicators with corresponding indicators of Faridabad District (CRHSP Ballabgarh belongs to Faridabad District). The District Level Household and Facility Survey, conducted by the Ministry of Health and Family Welfare Government of India (2007-08) reported a much lower coverage for all the indicators in the rest of the district as compared to CRHSP indicators (Table 4) [24]. Therefore there is no doubt that a computerized HMIS definitely will help in better service delivery program planning and better monitoring.

Having a functional demographic database has other advantages as well. Very often, the government, under different programs asks health functionaries to carry out needs assessment surveys. Many of these would be obviated if we switch over to an electronic system. It is necessary that these advantages are weighed against the cost and other inputs required to put such a system in place on a scale of a country like India; we have seen that the actual cost can be easily recovered within two years if the system is functional.

### Limitations of the study

One limitation of the study is that the investigators who were a part of the system being evaluated themselves planned and carried out the study. Though efforts were made to carry out interviews in a neutral unbiased manner, the possibility of interviewer bias cannot be ruled out. Secondly, the responses were recorded as narrated by the participants and their estimate of time taken for various activities was subjective. A time motion study would have been an ideal method to actually document the time spent by the health workers in both the groups. But due to logistic reasons this could not be carried out. The study did not look into issues of HMIS as a tool for organizational development. Another limitation is that the comparison with non-AIIMS HMIS was done only at the level of the health workers of non-AIIMS PHC. This provided us with information about the perceived benefits of the end users only. An interview with the medical officers of non-AIIMS PHCs would have given us more insights into the issues from the program managers' perspective.

The *future challenges *can be at two levels: intramural and extramural. The first one is to have sufficient intramural technical support to continue to address programmatic changes as new programs are added like that of chronic non-communicable diseases. Realization of research potential of a longitudinal population database is yet to happen. Extramurally, the challenge is to replicate this system in routine PHCs of state governments so as to enable a full evaluation of its implementation in a routine health system of the government as opposed to a health system run by an institution. We have not yet met with success on efforts to roll-out this HMIS more widely. While many people have shown interest in the HMIS, the interest has not yet been translated into action. The main concern is related to technical aspects of computerization till Community Health Centre (secondary level) or Primary Health Centre level. In 2005, the National Rural Health Mission was launched by the Government of India. This is a flagship program of the Government to strengthen the health system of the country. In this program, computerization is being taken up in a serious way, and we hope that inputs from our experience will be important for a large scale implementation. Once it is scaled up, the organizational and behavioral challenges may become more important.

## Conclusion

In summary, maintaining a good HMIS is essential for an effective health system in any country. However we have seen that in India and other developing countries this component is weak and therefore there is often a lack of good quality data and inefficient utilization of resources. Our experience at Ballabgarh shows that computerizing the HMIS using a name based system is feasible and has several advantages. It improves effectiveness, efficiency, saves resources and is a flexible framework. The major advantage of computerization has been in saving of time of health workers in record keeping and report generation. The initial capital costs of computerization can be recovered within two years of implementation if the system is fully operational. Computerization has enabled implementation of a good system for service delivery, planning, monitoring and supervision. In future, it can also provide a platform for convergence of different services related to social sectors.

## Competing interests

The authors declare that they have no competing interests.

## Authors' contributions

KA conceived of the study, and participated in its design and coordination and helped to draft the manuscript. NB participated in the design of the study, data collection and drafting the manuscript. KY and VG assisted in data collection and statistical analysis. SS contributed in designing the software and assisted in implementation of the program. All authors read and approved the final manuscript.

## Appendix: Interview schedule used for indepth interviews

### A. Interview Schedule for Health workers (using manual HMIS, non-computerized)

#### Introduction

Introduction of interviewer

Confirmation of interviewees' identification details

Explanation of the research objectives

Explanation of confidentiality and taking informed consent

#### Questions

1. I would like to start by asking you about details regarding the record keeping/maintaining registers that you are required to do as part of your job as a health worker?

Prompts:

How many registers do you have to maintain?

When do you fill up the registers?

How much time do you take to fill up the registers?

What problems do you face if any, while filling/completing the registers?

2. How do you estimate the target population for the different services that you are required to deliver to the community in your sub-centre? For eg total number of children eligible for immunization, number of antenatal women, etc.

Prompts:

Do you face any difficulty in estimating the target population?

How accurate is this estimate in your opinion? If possible can you express the accuracy/completeness of estimates of target population in percentages?

3. How do you calculate the coverage of different services being delivered by you as part of your job as health worker? For eg. Immunisation coverage, percentage of antenatal women given Iron tablets, etc.

Prompts:

How exactly do you calculate the immunization coverage rates? How do you calculate the numerator and denominator for the same?

Do you face any difficulty in calculating the immunization coverage rates? If yes, what?

How do you identify backlogs of unimmunized children?

4. How do you identify the new births and deaths occurring in the community covered by your subcentre?

Prompts:

In your opinion how complete are the birth and death registration?

*Do you face any difficulty in updating new births and deaths? If yes please explain*.

5. Do you carry out a census or any other similar activity in your area to update your records? If yes, how frequently?

Prompts:

When is the census carried out?

How much time does it take to complete the census in your subcentre?

How do you update your records after the census? How much time does it take for updating the records after the census?

6. Do you prepare a monthly report of activities/services carried out by you as part of your work every month? If yes is the report as per the prescribed format?

Prompts:

How do you prepare the monthly report? From which source is the information required for monthly report collected?

How much time does it take to prepare a monthly report?

Do you face any problems in compiling the monthly report? If yes, what?

7. Do you receive any feedback from your supervisors/higher authorities on the work done by you during the month?

Prompts:

Do you get any feedback on the monthly report that you submit?

Do you think the feedback that you receive (if you receive any) is useful?

### B. Interview Schedule for Health workers (using computerized HMIS)

Same as 1-7 above except for the following one additional question:

8. Do you think computerization of HMIS/record keeping has made your work easier/simpler or more difficult? Please explain how?

Prompts:

Please give the specific features of computerized HMIS that have made your work easier or difficult?

*Please explain how the identified feature has helped you or impeded your work*.

## Source of funding

All India Institute of Medical Sciences, New Delhi, India. The funding body, does not have any role in the collection, analysis, and interpretation of data, in the writing of the manuscript and in the decision to submit the manuscript for publication.

## Pre-publication history

The pre-publication history for this paper can be accessed here:

http://www.biomedcentral.com/1472-6963/10/310/prepub

## Supplementary Material

Additional file 1 **Table S1: The framework for evaluation of Ballabgarh HMIS**. Click here for file
