# Supplementary material for: Evaluation of computerized health management information system for primary health care in rural India
Source: BMC Health Serv Res. 2010 Nov 16;10:310. doi: 10.1186/1472-6963-10-310 (PMC2996385; doi:10.1186/1472-6963-10-310)
Supplement: Additional file 1 — Table S1: The framework for evaluation of Ballabgarh HMIS. [file 1472-6963-10-310-S1.DOC]

1. Inputs:
   1. Technical Support –
      1. Hardware maintenance
      2. Software maintenance
   2. Organizational
      1. Training
      2. Finances
      3. Sustainability
2. Processes
   1. Data Flow.
   2. Data Security procedures
3. Outputs:
   1. Data quality
   2. Information Use
4. Outcomes (Improved system performance)
   1. Users’ perspective of advantages and disadvantages.
   2. Program coverage indicators
